# EduFairBench: reproducible evaluation of large language models for educational assessment

**DOI:** 10.3389/frai.2026.1933445

**Published:** 2026-08-28

**Authors:** William Villegas-Ch, Aracely Mera-Navarrete, Fernando Zúñiga-Tello, Rommel Gutiérrez

**Affiliations:** 1 Facultad de Ingenierías y Ciencias Aplicadas, Universidad de Las Américas, Quito, Ecuador; 2 Departamento de Sistemas, Universidad Internacional del Ecuador, Quito, Ecuador; 3 Facultad de Comunicación y Artes Audiovisuales, Universidad de Las Américas, Quito, Ecuador

**Keywords:** automated essay scoring, educational assessment, feedback quality, large language models, reproducibility, short-answer grading, uncertainty

## Abstract

Large language models (LLMs) are increasingly used to evaluate open-ended educational responses. However, their performance is often assessed using aggregate metrics that provide limited insight into prediction stability, uncertainty, error patterns, and feedback quality. This study presents EduFairBench, a reproducible evaluation protocol designed to characterize LLM behavior across short-answer assessment and automated essay scoring using open educational benchmarks. The protocol combines repeated inference, majority-vote consolidation, uncertainty estimation, error analysis, and structural evaluation of generated feedback within a unified experimental framework. Experiments were conducted on SciEntsBank, Beetle, and ASAP2, comprising 2,000 student responses and 10,000 independent LLM inferences. The results showed moderate predictive agreement with human assessment while revealing substantial differences between nominal and ordinal evaluation tasks. Repeated inference demonstrated high internal stability across benchmarks, although systematic errors remained in semantically adjacent categories, indicating that prediction consistency does not necessarily imply correctness. Feedback quality varied by task type, with longer textual contexts yielding more specific and pedagogically structured explanations. These findings demonstrate that evaluating educational LLMs requires complementary analyses beyond conventional performance metrics. EduFairBench provides a reproducible methodology for jointly analyzing predictive performance, robustness, uncertainty, and feedback quality, providing a comprehensive methodological framework for the rigorous evaluation of LLM-based educational assessment systems.

## 1 Introduction

Large Language Models (LLMs) have significantly expanded the ability of artificial intelligence (AI) to interpret, generate, and analyze natural language, enabling their integration into domains where decisions depend on complex textual information. Their rapid adoption has enabled new applications in research, industry, and education while also raising important questions about the reliability, transparency, and responsible use of AI-generated decisions (Dwivedi et al., 2023; Southworth et al., 2023). As LLMs become increasingly involved in tasks that influence human decision-making, the central challenge is no longer whether these models can generate plausible responses, but under which conditions their outputs can be considered sufficiently reliable, reproducible, and suitable for real-world applications.

Educational assessment represents one of the most demanding settings for this challenge. LLMs have demonstrated considerable potential for tutoring, content generation, automated assessment, and personalized feedback because they can process open-ended responses and produce natural-language explanations. At the same time, recent studies emphasize that their educational adoption requires rigorous empirical validation, transparent evaluation procedures, and systematic evidence regarding both their capabilities and limitations before they can be incorporated into high-impact assessment processes (Grassini, 2023; Kohnke et al., 2023; Sallam, 2023). Consequently, research has progressively shifted from exploring what LLMs can do to developing reliable methodologies for evaluating their behavior in educational settings.

The automatic assessment of open-ended responses illustrates this challenge particularly well. Unlike closed-ended tasks, evaluating short answers and essays requires interpreting heterogeneous linguistic evidence, distinguishing different levels of conceptual understanding, and producing judgments comparable to those of human evaluators. Although recent studies report competitive predictive performance, evaluation is still commonly based on aggregate metrics obtained from a single model execution. This practice provides limited insight into the reproducibility of predictions, the stability of repeated inferences, the uncertainty associated with model decisions, or the reliability of the generated feedback.

These limitations extend beyond the choice of evaluation metrics. Two models with similar overall performance may exhibit substantially different behaviors regarding error distribution, prediction stability, confidence calibration, or feedback quality. Likewise, highly consistent predictions do not necessarily correspond to correct decisions, and well-structured explanations may accompany incorrect classifications. Consequently, current evaluation practices provide only a partial characterization of LLM behavior in educational assessment. Existing studies rarely integrate predictive performance, inference stability, uncertainty quantification, error patterns, and feedback quality into a single reproducible evaluation protocol, making it difficult to compare findings across studies or distinguish model behavior from artifacts introduced by experimental design. This methodological gap motivates the work presented in this paper: to establish a reproducible protocol capable of providing a multidimensional characterization of LLM behavior in educational assessment.

To address this need, this paper introduces *EduFairBench*, a reproducible evaluation protocol designed to systematically characterize the behavior of large language models in open-ended educational assessment using publicly available educational benchmarks. Rather than proposing a new assessment model or optimizing predictive performance, EduFairBench provides a reusable experimental framework that integrates repeated inference, prediction consolidation, uncertainty estimation, error-pattern analysis, and structural evaluation of generated feedback within a fully traceable workflow. The protocol is model-agnostic and can be applied to different language models without modifying the underlying evaluation methodology, facilitating consistent and reproducible experimental comparisons across systems. The novelty of EduFairBench is methodological rather than algorithmic: it does not claim majority voting, bootstrap intervals, uncertainty indices, or error analysis as new techniques, but combines them into a single auditable protocol in which every reported metric is linked to a preserved dataset instance, prompt version, model execution, parsed output, and consolidated prediction. The resulting framework enables multiple complementary dimensions of model behavior to be examined using a unified and reproducible experimental methodology.

The proposed framework is intended to promote reproducible evaluation practices across different language models, educational benchmarks, and assessment scenarios. By integrating predictive performance, robustness, uncertainty, and the structural quality of generated feedback, EduFairBench provides a methodological basis for more consistent comparisons across studies while providing methodological evidence to support transparent, auditable, and reproducible AI-assisted educational assessment.

## 2 Literature review

The incorporation of large language models (LLMs) has transformed educational assessment by shifting the focus from specialized scoring systems toward foundation models capable of interpreting, reasoning about, and generating responses across domains without task-specific training. This transition expands the possibilities for automatically assessing open-ended responses, including short-answer classification, automated essay scoring (AES), criterion-based grading, and personalized feedback generation. At the same time, it intensifies long-standing concerns about validity, transparency, fairness, and traceability in automated educational decisions (Kasneci et al., 2023; Yan et al., 2024; Emirtekin, 2025).

Recent reviews show that LLM-powered assessment has been applied to essay scoring, reading comprehension, language learning, computer science, and other open-response contexts, but also report substantial variation in human–AI agreement, reliability, hallucination risk, and methodological documentation (Yan et al., 2024; Emirtekin, 2025). In this literature, the central issue is not only whether LLMs can reproduce human scores, but whether their behavior can be evaluated under protocols that disclose datasets, prompt templates, inference settings, repeated executions, and uncertainty indicators. This requirement is especially important because LLM outputs remain sensitive to prompting, temperature, model version, and response-format constraints.

This concern is consistent with broader reviews of ChatGPT and artificial intelligence in higher education, which emphasize that educational applications require transparent reporting, institutional policies, ethical safeguards, and explicit attention to assessment integrity before they are adopted in consequential learning environments (Lo, 2023; Crompton and Burke, 2023; Chan, 2023; Nguyen et al., 2023).

In automated essay assessment, recent empirical studies have examined GPT-based scoring, prompt strategies, intra-rater reliability, and alignment with human judgments. Kim (2025) showed that GPT-4 scoring behavior depends on rubric detail, rationale requirements, scoring examples, and repeated ratings. Similarly, Manning et al. (2025) compared human raters with ChatGPT-based essay scoring and found that alignment with human evaluation cannot be assumed even when model outputs appear consistent. Complementary work has proposed hybrid AES architectures that combine LLM-derived representations with linguistic features, reinforcing the need to evaluate both predictive accuracy and construct validity (Atkinson and Palma, 2025).

Recent work has also moved beyond single-model evaluation. Saez et al. (2026) evaluated multiple state-of-the-art LLMs for AI-assisted grading in higher education and emphasized that model selection should consider grading accuracy, feedback quality, semantic alignment, and cost. Shin (2026) proposed an AI-enhanced assessment framework for middle-school written responses that incorporated scoring rationales, uncertainty detection, aggregation, and teacher review. These studies support the view that model performance must be interpreted jointly with uncertainty, reproducibility, and human oversight rather than as a single aggregate score.

Feedback generation represents another methodological frontier. LLMs can produce explanations and recommendations, but the educational usefulness of such feedback cannot be inferred directly from classification accuracy. Recent evaluations of AI-assisted grading and automated assessment emphasize that feedback should be assessed for specificity, constructiveness, relevance, and alignment with the assigned score (Saez et al., 2026; Emirtekin, 2025). This distinction is important because a coherent explanation may still accompany an incorrect prediction, while a small ordinal scoring deviation may coexist with useful formative guidance.

The same literature also warns that LLM adoption in education is shaped by practical constraints, academic-integrity risks, and uneven pedagogical readiness; therefore, reproducible evaluation frameworks should document not only model performance but also the governance and reliability conditions under which automated feedback and scoring are produced (Farrokhnia et al., 2024; Cotton et al., 2024; Tlili et al., 2023).

Collectively, the literature reveals six recurring limitations: incomplete dataset documentation, insufficient reporting of prompt design and inference parameters, limited repeated-inference analysis, scarce uncertainty-aware evaluation, weak characterization of systematic error mechanisms, and limited separation between predictive performance and feedback quality. EduFairBench addresses these gaps through a reproducible, model-agnostic protocol that integrates dataset auditing, prompt-version traceability, repeated inference, majority-vote consolidation, uncertainty estimation, semantic error analysis, and structural feedback evaluation within a unified methodological framework.

## 3 Materials and methods

### 3.1 Datasets, unit of analysis, and traceability

The experimental protocol was designed around three educational benchmarks representing two complementary paradigms of automated assessment: nominal short-answer grading and ordinal essay scoring. This distinction determined the unit of analysis, the evaluation metrics, and the methodological workflow adopted throughout the study. SciEntsBank and Beetle were used for short-answer assessment, whereas ASAP2 was employed for automated essay scoring. Accordingly, the experimental unit corresponded to the student–question pair in the short-answer benchmarks and the student–prompt pair in ASAP2, preserving the distinction between nominal and ordinal assessment throughout the entire experimental workflow.

The integrated benchmark combines educational scenarios with substantially different structural characteristics under a common and reproducible experimental protocol. Following data normalization, SciEntsBank contributed 10,804 responses distributed across 195 independent questions, Beetle contributed 5,199 responses associated with 42 engineering problems, and ASAP2 contributed 24,728 essays corresponding to seven writing prompts. These datasets represent complementary assessment paradigms rather than variations of the same task. Whereas the short-answer benchmarks require discrimination among mutually exclusive semantic categories, ASAP2 requires reproducing an ordinal representation of writing quality. Integrating both task families makes it possible to evaluate language model behavior across heterogeneous educational settings while maintaining a unified experimental methodology.

Dataset integrity was verified before any experimental stage was conducted. The audit identified no missing values in the fields required to preserve the correspondence among student responses, human references, and benchmark metadata. Preprocessing was restricted to field harmonization, persistent identifier assignment, benchmark-specific label-name normalization for analysis, response-length computation, and validation of required fields. Original student responses, human labels, human scores, questions, prompts, and benchmark metadata were preserved. No labels or scores were imputed, corrected, reweighted, or synthetically augmented, and no class rebalancing was applied. The methodological summary in Table 1 reports the corpus size, final sample, human reference scale, full-corpus distribution, and sampling variables used for each benchmark.

**Table 1:** Dataset structure and sampling variables used in the experimental protocol.

Benchmark	Full corpus	Final sample	Human reference	Full-corpus distribution	Sampling variables
ASAP2	24,728	1,000	Scores 1–6	3: 36.5%; 2: 27.7%; 4: 22.5%; 1: 7.1%; 5: 5.5%; 6: 0.8%	Prompt, score, response length
Beetle	5,199	500	Five labels	Correct: 42.0%; contradictory: 27.0%; partial: 23.1%; non-domain: 5.0%; irrelevant: 2.9%	Question, human label
SciEntsBank	10,804	500	Five labels	Correct: 41.3%; irrelevant: 24.6%; partial: 24.0%; contradictory: 9.6%; non-domain: 0.4%	Question, human label

Traceability was ensured through persistent identifiers assigned to every experimental instance from the original datasets to the consolidated results. All artifacts generated during data auditing, normalization, sampling, repeated inference, prediction consolidation, and statistical analysis were documented using version-controlled manifests together with SHA-256 cryptographic hashes. This mechanism guarantees that every metric, table, figure, and representative case reported in the following sections can be reconstructed directly from the normalized datasets, the final experimental samples, and the recorded model outputs without introducing transformations outside the controlled experimental workflow.

**Figure 1:**
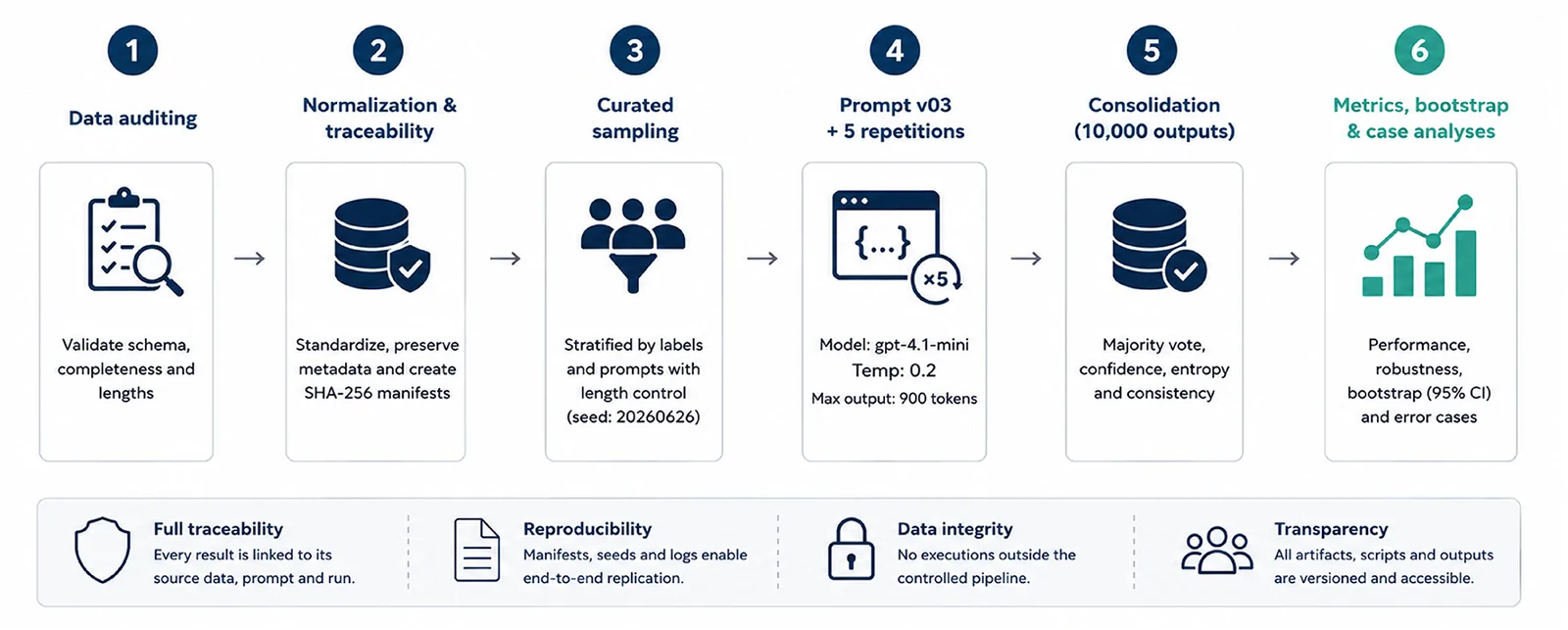
Reproducible workflow of the EduFairBench evaluation protocol, illustrating the six sequential stages from data auditing to statistical analysis and feedback evaluation.

Figure 1 summarizes the complete EduFairBench workflow through six sequential stages: data auditing, normalization and traceability, experimental sampling, repeated inference, prediction consolidation, and statistical analysis. Together, these stages preserve the correspondence between the original datasets, every model execution, and the consolidated experimental outcomes, ensuring complete traceability throughout the evaluation process. The following subsections describe each stage in detail, including the sampling strategy, inference protocol, evaluation metrics, and statistical procedures that collectively ensure the reproducibility of the proposed methodology.

### 3.2 Final sampling and LLM execution protocol

The experimental sampling strategy was designed to preserve the representativeness of each educational benchmark while maintaining the original composition of the assessment tasks. The final experimental design included 1,000 instances from ASAP2, 500 from Beetle, and 500 from SciEntsBank, providing a computationally feasible evaluation set without altering the conceptual diversity or the distribution of human annotations present in the original corpora.

The sampling procedure was adapted to the characteristics of each assessment family. For the short-answer benchmarks, proportional allocation according to the distribution of human labels was combined with a per-question coverage criterion to ensure representation of every assessment scenario. For ASAP2, sampling was performed using strata defined by the combination of writing prompt and human score, incorporating essay-length intervals to prevent a single region of the textual space from dominating the final sample. A fixed random seed (20260626) was used throughout the sampling procedure to ensure complete reproducibility of the selected experimental sample.

The sampling audit confirmed that the original structure of the three educational benchmarks was preserved. The final sample retained all 195 independent questions from SciEntsBank, the 42 engineering problems from Beetle, and the seven writing prompts from ASAP2 while maintaining the original distribution of human annotations within each educational domain. The audit results were recorded exclusively as a reproducibility control mechanism and were not used to modify any subsequent stage of the experimental protocol.

Model inference was performed using two task-specific instruction templates. For short-answer assessment, the prompt required each student response to be classified into exactly one of the five benchmark categories (*correct, contradictory, partially_correct_incomplete, irrelevant*, and *non–domain*), emphasizing semantic equivalence with the reference response and the explicit distinction among contradictory, irrelevant, and non–domain answers. For ASAP2, the template requested an ordinal score ranging from 1 to 6 together with structured pedagogical feedback describing strengths, weaknesses, recommendations for improvement, and a justification for the assigned score. In both task families, model outputs were required to conform to predefined JavaScript Object Notation (JSON) schemas that were automatically validated before entering the subsequent stages of the experimental pipeline.

Prompt development was restricted to task alignment, schema compliance, and pilot validation of parseable outputs. The definitive experiment used a single frozen prompt version (v03) composed of two final task-specific templates: one for short-answer classification and one for ordinal essay scoring. Earlier prompt refinements were treated as pilot calibration steps and were not included as experimental conditions, prompt-comparison arms, or optimization procedures in the final analysis. Table 2 summarizes the final prompt configuration, inference parameters, execution protocol, and traceability fields adopted in the definitive experiment.

**Table 2:** Prompt configuration and inference settings adopted in the definitive experiment.

Component	Final setting
Prompt version	v03, fixed before definitive execution
Prompt templates	Two templates: short-answer classification and ordinal essay scoring
Prompt variations in final analysis	None; prompt variants were not compared as experimental conditions
Output format	Validated JSON with task-specific score or label, confidence, feedback, and audit rationale fields
Model	GPT-4.1-mini
Temperature	0.2
Maximum output length	900 tokens
Repeated executions	Five independent inferences per experimental instance under identical settings
Traceability fields	prompt_version, prompt_instance_id, execution_id, model, temperature, max_output_tokens, parse_status

The empirical execution reported in this study used GPT-4.1-mini as the evaluated model. The protocol itself does not depend on this model: the same dataset auditing, prompting, repeated-inference, consolidation, validation, and statistical stages can be applied to alternative LLMs without changing the evaluation design.

The repeated inference strategy can be formally represented by Equation 1, where each experimental instance generates a set of five independent model responses,


$$\begin{array}{c} R_{i}=\left\{r_{i}^{\left(1\right)},r_{i}^{\left(2\right)},r_{i}^{\left(3\right)},r_{i}^{\left(4\right)},r_{i}^{\left(5\right)}\right\}, \end{array}$$ (1)

where $r_{i}^{\left(k\right)}$ denotes the prediction produced during the *k*-th repeated inference for experimental instance *i*.

The consolidated prediction used throughout the experimental workflow was obtained using the majority-vote operator defined in Equation 2,


$$\begin{array}{c} {ŷ}_{i}=\arg\underset{c}{\max}n\left(c|R_{i}\right), \end{array}$$ (2)

where *n*(*c*|*R*_*i*_) denotes the number of occurrences of category *c* among the repeated predictions associated with instance *i*.

This consolidation strategy separates the variability inherent to repeated inference from the final prediction used to compute predictive performance, robustness indicators, uncertainty estimates, and feedback quality metrics, thereby ensuring that all subsequent analyses are based on a single, reproducible decision for each experimental instance.

The complete execution and prediction consolidation procedure implemented in EduFairBench is summarized in Algorithm 1. The algorithm describes the reproducible workflow followed for every benchmark and experimental instance, from repeated inference to the generation of the consolidated prediction and the corresponding traceability metadata.

Algorithm 1 **EduFairBench repeated inference and prediction consolidation protocol**

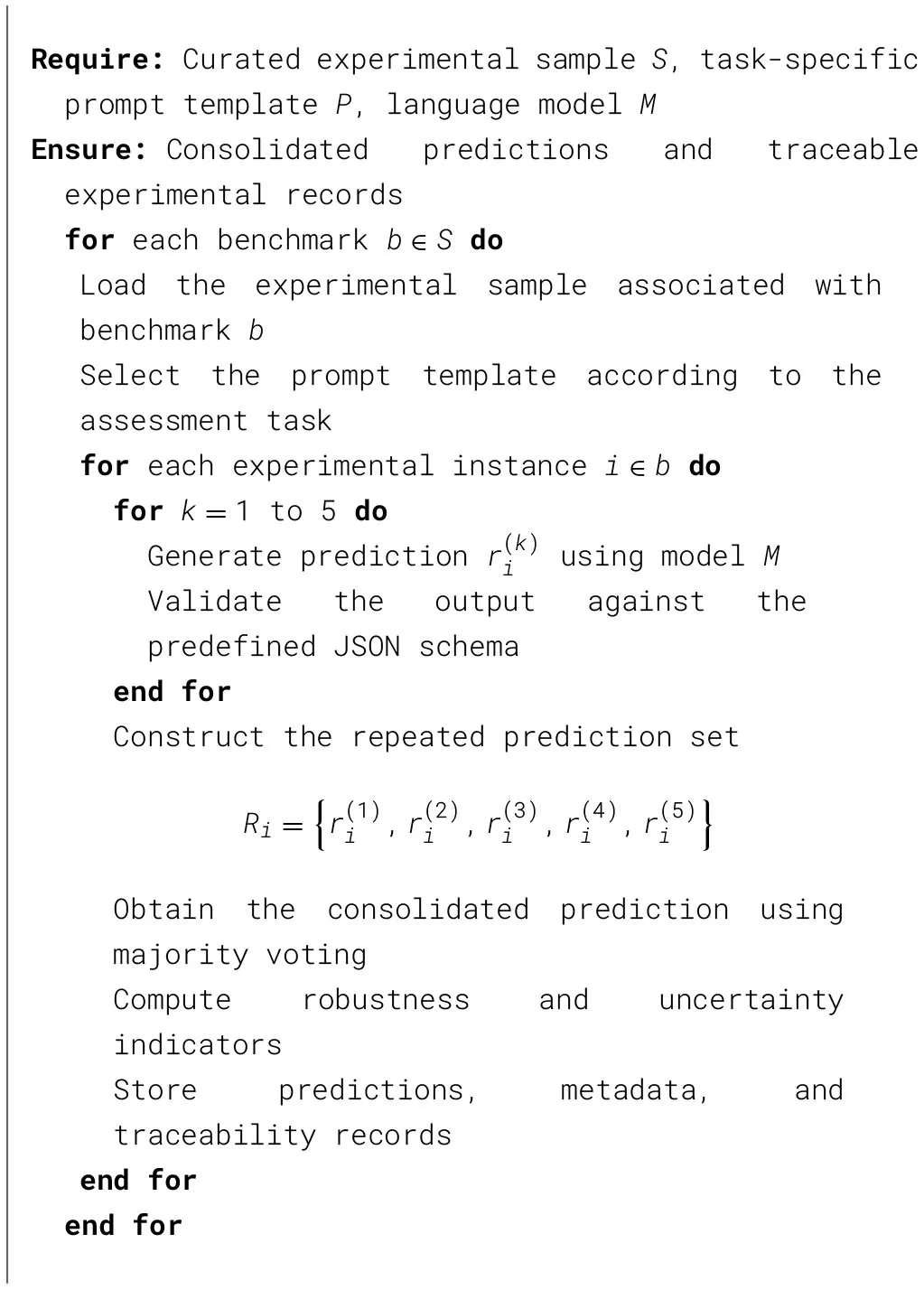

### 3.3 Metrics, uncertainty estimation, and statistical protocol

Performance evaluation was defined according to the characteristics of each assessment task, avoiding the interpretation of nominal and ordinal outcomes under a common evaluation criterion. For the short-answer benchmarks, the consolidated prediction obtained through majority voting was compared with the corresponding human annotation using Accuracy, Macro-F1, Weighted-F1, macro precision, macro recall, and Cohen's Kappa coefficient. Together, these metrics characterize overall predictive performance, class balance, and nominal agreement beyond chance. In addition, class-specific performance metrics were computed for each benchmark category (*correct, contradictory, partially_correct_incomplete, irrelevant*, and *non–domain*) to identify asymmetric prediction behavior across semantic classes.

Essay assessment required a different set of evaluation measures because of the ordinal nature of human scores. Consolidated predictions were therefore compared with the reference annotations using the Mean Absolute Error (MAE), Root Mean Squared Error (RMSE), Pearson and Spearman correlation coefficients, and the Quadratic Weighted Kappa (QWK). Among these measures, QWK was adopted as the primary agreement metric because it explicitly preserves the ordinal structure of the rating scale while assigning larger penalties to disagreements between distant score categories than to disagreements between adjacent levels. The coefficient is defined by Equation 3,


$$\begin{array}{c} QWK=1-\frac{\underset{i,j}{\sum }w_{ij}O_{ij}}{\underset{i,j}{\sum }w_{ij}E_{ij}}, \end{array}$$ (3)

where *O*_*ij*_ denotes the observed agreement matrix, *E*_*ij*_ the agreement matrix expected by chance, and *w*_*ij*_ the penalty assigned to each ordinal disagreement. This formulation quantifies the agreement between human ratings and model predictions while explicitly accounting for the magnitude of ordinal prediction errors.

Uncertainty associated with the primary evaluation metrics was estimated using a non-parametric bootstrap procedure with 2,000 resamples. The bootstrap was applied to the consolidated predictions obtained through majority voting, thereby preserving the experimental unit defined by the protocol. All resampling procedures were executed within the same reproducible computational environment adopted throughout EduFairBench. Empirical bootstrap distributions were subsequently used to estimate 95% confidence intervals (CIs) for the primary metrics reported for each benchmark, providing robust uncertainty estimates without assuming parametric distributions of the underlying data.

The statistical protocol was intentionally restricted to procedures compatible with the experimental design. The study evaluated a single language model using one prompting configuration and a single reproducible inference protocol; therefore, no paired experimental conditions or multiple treatment groups were available to justify inferential procedures such as the Wilcoxon signed-rank test, the Friedman test, the Nemenyi *post-hoc* procedure, or between-group effect-size analyses. Consequently, statistical characterization relied on the complementary use of predictive performance metrics, category-level analyses, robustness indicators, and bootstrap-based confidence intervals. This combination provides a statistically consistent framework for quantifying both model performance and the uncertainty associated with the reported estimates while remaining fully aligned with the reproducible design of EduFairBench.

### 3.4 Error analysis, robustness, and feedback evaluation

The interpretation of model outputs was organized around four complementary dimensions: prediction behavior, inference robustness, structural quality of LLM-generated feedback, and qualitative analysis of representative cases. This multidimensional organization enables the evaluation of language models from perspectives that cannot be inferred solely from aggregate performance metrics, distinguishing predictive correctness from inference stability and the pedagogical usefulness of the generated feedback.

Prediction behavior was analyzed separately for nominal and ordinal assessment tasks because each task family is governed by different decision mechanisms. For the short-answer benchmarks, class-specific performance metrics and transitions between human annotations and consolidated predictions were examined to identify semantic boundaries associated with the highest classification difficulty. For ASAP2, the analysis focused on shifts between human and predicted scores together with the distribution of residual and absolute errors across the ordinal rating scale. Treating these task families independently avoids interpreting fundamentally different error mechanisms under a common criterion and facilitates a more precise characterization of model behavior.

Inference robustness was evaluated independently of predictive performance using the five repeated executions available for every experimental instance. Exact consistency, majority agreement, prediction entropy, and mean confidence were computed for all benchmarks. For ASAP2, the variance of predicted scores was additionally calculated to characterize the dispersion of ordinal predictions. These indicators do not measure agreement with the human reference but rather quantify the ability of the language model to reproduce stable predictions under identical experimental conditions.

The uncertainty associated with repeated inference was quantified using Shannon entropy, formally defined by Equation 4,


$$\begin{array}{c} H_{i}=-\underset{k}{\sum }p_{ik}\log_{2}\left(p_{ik}\right), \end{array}$$ (4)

where *p*_*ik*_ denotes the proportion of repeated inferences assigning category or score *k* to experimental instance *i*. Lower entropy values indicate a stronger concentration of predictions and therefore greater stability across repeated executions.

Complementarily, exact consistency was defined by Equation 5,


$$\begin{array}{c} C_{i}=1\left(r_{i}^{\left(1\right)}=r_{i}^{\left(2\right)}=r_{i}^{\left(3\right)}=r_{i}^{\left(4\right)}=r_{i}^{\left(5\right)}\right), \end{array}$$ (5)

where **1**(·) denotes the indicator function, taking the value 1 only when the five repeated inferences produce an identical prediction. Together, entropy and exact consistency provide complementary descriptions of inference robustness by characterizing both the overall concentration of predictions and the existence of complete agreement across repeated executions.

The structural quality of LLM-generated feedback was quantified using seven objective indicators extracted directly from the validated JSON outputs: specificity, constructiveness, actionability, clarity, completeness, presence of suggestions, and presence of explanations. An overall feedback quality index was computed as the arithmetic mean of these indicators. This index was interpreted exclusively as a reproducible structural measure of generated feedback and was not intended to replace pedagogical evaluation performed by human experts. The feedback rubric was therefore operational rather than human-rated: each indicator was computed from predefined textual and structural criteria applied to the model output fields. No teachers, students, or external evaluators scored the generated feedback; consequently, no multiple-rater design was implemented, and interrater agreement did not apply to this component of the protocol.

Finally, qualitative interpretation was based on representative cases selected according to predefined and reproducible criteria from the consolidated predictions. The selected cases included correct classifications, severe errors, ambiguous responses, high-confidence misclassifications, correct predictions with comparatively low confidence, and examples illustrating both high and low structural feedback quality. These cases served exclusively as explanatory evidence for the aggregate quantitative findings and did not influence the calculation of any performance metric or statistical indicator reported in the study. These four analytical components constitute the multidimensional evaluation stage of EduFairBench, enabling the protocol to characterize predictive performance, robustness, uncertainty, and feedback quality within a unified and fully reproducible experimental framework.

## 4 Results

### 4.1 Benchmark characterization

The reliability of an educational LLM evaluation protocol depends on simultaneously preserving conceptual diversity, statistical representativeness, and inference stability throughout the experimental process. When one of these components is overemphasized, the observed differences may reflect characteristics of the benchmark itself rather than the underlying behavior of the evaluated language model. As summarized in Table 3, the three educational benchmarks represent clearly differentiated assessment scenarios. SciEntsBank provides the greatest conceptual diversity through a large number of independent questions, whereas Beetle concentrates a higher density of responses within a smaller collection of engineering concepts. In contrast, ASAP2 replaces short-answer classification with full essay assessment, where evaluation depends on integrating multiple arguments distributed throughout each document. Together, these benchmarks enable the analysis of language model behavior across educational settings that differ substantially in semantic variability and assessment complexity while preserving a common and reproducible experimental protocol.

**Table 3:** Structural characteristics of the educational benchmarks and experimental configuration adopted in EduFairBench.

Benchmark	Task	Full corpus	Sample	LLM inferences	Questions / Prompts	Consistency	Mean confidence
SciEntsBank	Short answer	10,804	500	2,500	195	0.908	0.881
Beetle	Short answer	5,199	500	2,500	42	0.886	0.884
ASAP2	Essay	24,728	1,000	5,000	7	0.925	0.851
Combined	Mixed	40,731	2,000	10,000	244	0.906	0.872

Experimental representativeness was preserved through a balanced sampling strategy that prevented the large differences in corpus size from influencing the subsequent evaluation. Although the three benchmarks differ substantially in scale, the final experimental protocol consisted of 2,000 instances and 10,000 independent LLM inferences, providing a homogeneous basis for comparing heterogeneous educational assessment tasks. Consequently, the differences discussed in the following sections reflect the behavior of the language model under equivalent experimental conditions rather than artifacts introduced by dataset size or sampling imbalance.

The robustness indicators reveal distinct patterns across assessment tasks. ASAP2 achieved the highest exact consistency (0.925), followed by SciEntsBank (0.908) and Beetle (0.886), despite representing the most linguistically complex benchmark included in the study. This finding suggests that inference stability is influenced not only by response length but also by the structure of the underlying evaluation criterion. Whereas ordinal essay scoring permits gradual transitions between adjacent performance levels, short-answer classification requires sharper semantic boundaries between conceptually similar categories, increasing the likelihood of prediction variability across repeated executions.

Mean confidence follows a different pattern from exact consistency. Beetle (0.884) and SciEntsBank (0.881) produced confidence levels that were both higher than those observed for ASAP2 (0.851), indicating that confidence and inference stability capture complementary properties of model behavior. While essay assessment generated highly reproducible predictions with comparatively lower confidence, short-answer assessment produced more confident predictions that nevertheless exhibited greater variability across repeated executions. This distinction further supports the need to evaluate robustness and confidence independently rather than treating them as interchangeable indicators of model reliability.

**Figure 2:**
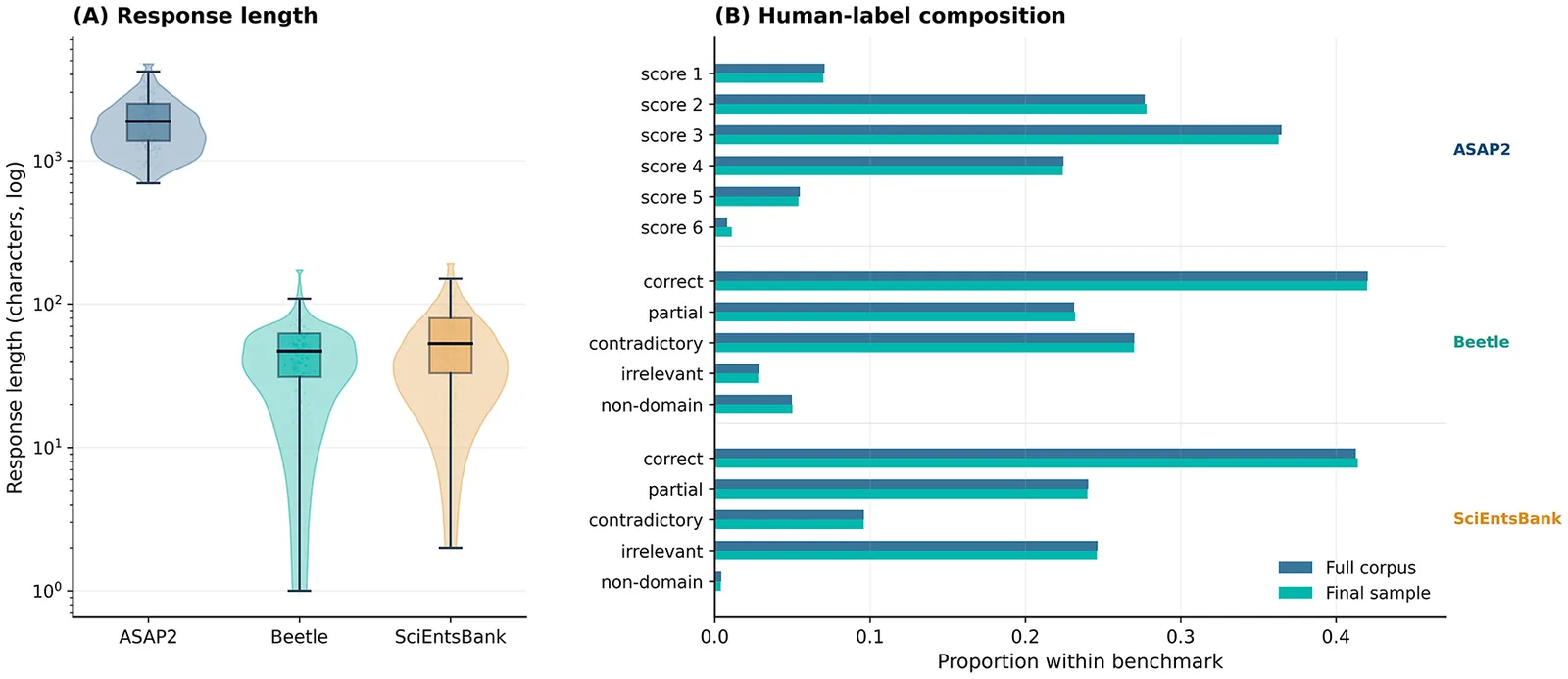
Statistical characterization of the experimental environment. **(A)** Distribution of response length by benchmark. **(B)** Comparison between the composition of the complete corpus and that of the final experimental sample.

Figure 2 summarizes the structural diversity of the experimental environment by jointly examining the linguistic characteristics of the three benchmarks and the preservation of the original corpus composition after sampling. As illustrated in Figure 2A, the response-length distributions exhibit an almost complete separation across datasets. SciEntsBank and Beetle are dominated by short textual responses in which the evaluation depends on subtle semantic distinctions expressed through only a few linguistic units. In contrast, ASAP2 shifts the distribution toward substantially longer texts, requiring the model to integrate argumentative coherence, discourse organization, and evidence distributed throughout the essay. These structural differences provide a complementary explanation for the distinct robustness and confidence patterns observed across the three educational assessment scenarios.

Figure 2B shows that the sampling procedure preserves the original distribution of human annotations across all three educational benchmarks. The relative frequencies observed in the final experimental sample closely match those of the complete corpora for every category, indicating that the reduction in sample size did not distort the statistical composition of the original datasets. Consequently, the comparative analyses presented in the following sections can be interpreted as differences arising from the language model's behavior under distinct educational assessment scenarios rather than from biases introduced during sample construction. This result confirms that the sampling strategy preserves the representativeness required for the reproducible evaluation protocol implemented by EduFairBench.

### 4.2 Overall model performance

Overall performance was analyzed separately for short-answer classification and automated essay scoring because these assessment tasks rely on fundamentally different decision mechanisms and evaluation criteria. Although both aim to reproduce human judgment, nominal and ordinal predictions require different performance measures and therefore cannot be interpreted under a common metric. Accordingly, model performance was first examined within each assessment family before conducting comparisons across educational benchmarks.

As summarized in Table 4, ASAP2 achieved a Quadratic Weighted Kappa (QWK) of 0.462 (95% CI: 0.420–0.500), indicating moderate ordinal agreement between the language model and human evaluation. Considering the Mean Absolute Error (MAE) together with QWK provides a more complete interpretation of ordinal performance. The corresponding 95% confidence interval for the MAE (0.611–0.690) indicates that the average prediction error remained below one score level throughout the rating scale. Together, these indicators suggest that prediction errors are concentrated predominantly between adjacent performance levels rather than arising from severe disagreements with human assessment.

**Table 4:** Overall predictive performance obtained for each educational benchmark.

Benchmark	Task	Primary	Estimate	95% CI	Secondary	Value	Interpretation
ASAP2	Essay	QWK	0.462	0.420–0.500	MAE	0.648	Moderate ordinal agreement with an average error below one score level.
Beetle	Short answer	Accuracy	0.608	0.564–0.650	Macro-F1	0.552	Higher nominal performance across semantic categories.
SciEntsBank	Short answer	Accuracy	0.580	0.534–0.622	Macro-F1	0.433	Greater difficulty distinguishing semantically adjacent categories.

The two short-answer benchmarks exhibit a distinct performance profile. Although Beetle (Accuracy = 0.608) and SciEntsBank (Accuracy = 0.580) achieve comparable levels of overall Accuracy, the difference becomes substantially larger when considering Macro-F1 (0.552 vs. 0.433). This divergence indicates that the two benchmarks impose different demands on the classification process. Whereas Beetle maintains relatively balanced performance across semantic categories, SciEntsBank exhibits a more pronounced degradation when all classes are weighted equally. This finding suggests that the greater conceptual coverage and linguistic diversity represented in SciEntsBank increase the difficulty of maintaining balanced predictive performance across all human annotation categories.

The comparison between the two benchmarks further demonstrates that model performance depends not only on the short-answer assessment format but also on the conceptual structure of the evaluated domain. The greater conceptual diversity represented by SciEntsBank increases the difficulty of discriminating among semantically adjacent categories, whereas Beetle presents a comparatively more homogeneous classification scenario. Consequently, Accuracy adequately summarizes overall predictive performance, whereas Macro-F1 reveals that the model's ability to distinguish among categories decreases as conceptual diversity increases. These observations confirm that global performance metrics alone are insufficient to characterize language model behavior in heterogeneous short-answer assessment tasks.

The confidence intervals further support these observations. For Beetle and SciEntsBank, the 95% confidence intervals associated with Accuracy partially overlap, indicating that the difference between the two benchmarks should not be interpreted as a clear separation in overall predictive performance. However, the larger divergence observed in Macro-F1 indicates that the principal difference lies in the model's ability to maintain balanced performance across categories with different frequencies and semantic complexity rather than in the overall proportion of correctly classified responses. For ASAP2, the confidence interval associated with QWK confirms a stable ordinal agreement, while the corresponding interval for MAE remains consistent with prediction shifts occurring predominantly between adjacent score levels.

To quantify the effect of majority-vote consolidation, the average performance of the five individual executions was compared with the consolidated prediction. Differences were small across benchmarks: in ASAP2, exact agreement changed from 0.441 to 0.436 and QWK from 0.464 to 0.462, while adjacent agreement changed from 0.917 to 0.919. In Beetle, Accuracy changed from 0.612 to 0.608 and Macro-F1 from 0.555 to 0.552. In SciEntsBank, Accuracy remained 0.580, and Macro-F1 changed from 0.433 to 0.433. Thus, the consolidated predictions produced nearly identical aggregate performance estimates while assigning one final prediction to each experimental instance.

### 4.3 Analysis of predictive behavior

#### 4.3.1 Errors in short-answer tasks

The reduction in predictive performance observed in short-answer assessment is not explained solely by the proportion of correctly classified responses but also by the model's ability to preserve stable semantic boundaries between conceptually adjacent categories. When these boundaries become ambiguous, aggregate measures such as Accuracy provide only a partial characterization of model behavior. As reported in Table 5, predictive performance varies substantially across evaluation categories. The *correct* class was the most stable category in both short-answer benchmarks, whereas the *non–domain* category achieved high F1 only in Beetle. In SciEntsBank, the *non–domain* class had extremely limited support and obtained an F1 score of 0.000, so it cannot be interpreted as a stable high-performing category. In contrast, lower performance in the *partially_correct_incomplete* and *contradictory* categories indicates greater difficulty distinguishing intermediate levels of conceptual understanding.

**Table 5:** Class-level predictive performance for the short-answer benchmarks.

Benchmark	Label	Instances	Precision	Recall	F1
Beetle	Contradictory	135	0.659	0.430	0.520
Beetle	Correct	210	0.731	0.776	0.753
Beetle	Irrelevant	14	0.105	0.143	0.121
Beetle	Non-domain	25	0.957	0.880	0.917
Beetle	Partially correct/incomplete	116	0.401	0.509	0.449
SciEntsBank	Contradictory	48	0.391	0.562	0.462
SciEntsBank	Correct	207	0.740	0.744	0.742
SciEntsBank	Irrelevant	123	0.776	0.423	0.547
SciEntsBank	Non-domain	2	0.000	0.000	0.000
SciEntsBank	Partially correct/incomplete	120	0.365	0.475	0.413

This degradation is particularly evident for the *partially_correct_incomplete* category, whose F1 score reaches only 0.449 in Beetle and 0.413 in SciEntsBank. Similarly, the *contradictory* category achieves F1 scores of 0.520 and 0.462, respectively. The simultaneous reduction in Precision and Recall suggests that the primary challenge is not insufficient representation of these classes but the instability of the semantic decision boundary separating partially correct, contradictory, and incomplete responses. These categories require subtle distinctions regarding both the amount and consistency of conceptual evidence contained in each student response.

The *irrelevant* category exhibits a markedly different behavior across the two benchmarks. Whereas SciEntsBank reaches an F1 score of 0.547, Beetle achieves only 0.121 despite using the same annotation category. This contrast suggests that irrelevance is determined not only by lexical content but also by the relationship between the student response and the assessment objective. In Beetle, many responses contain technically correct terminology while failing to address the question being asked, making it substantially more difficult for the model to distinguish genuinely irrelevant answers from responses containing partially useful conceptual evidence.

Taken together, these results indicate that the principal source of classification error in short-answer assessment is not the recognition of domain concepts but the consistent discrimination between semantically adjacent categories requiring different levels of conceptual evidence. This finding highlights that aggregate performance metrics alone are insufficient to explain model behavior and that class-level analyses are essential for identifying the semantic mechanisms underlying prediction errors.

**Figure 3:**
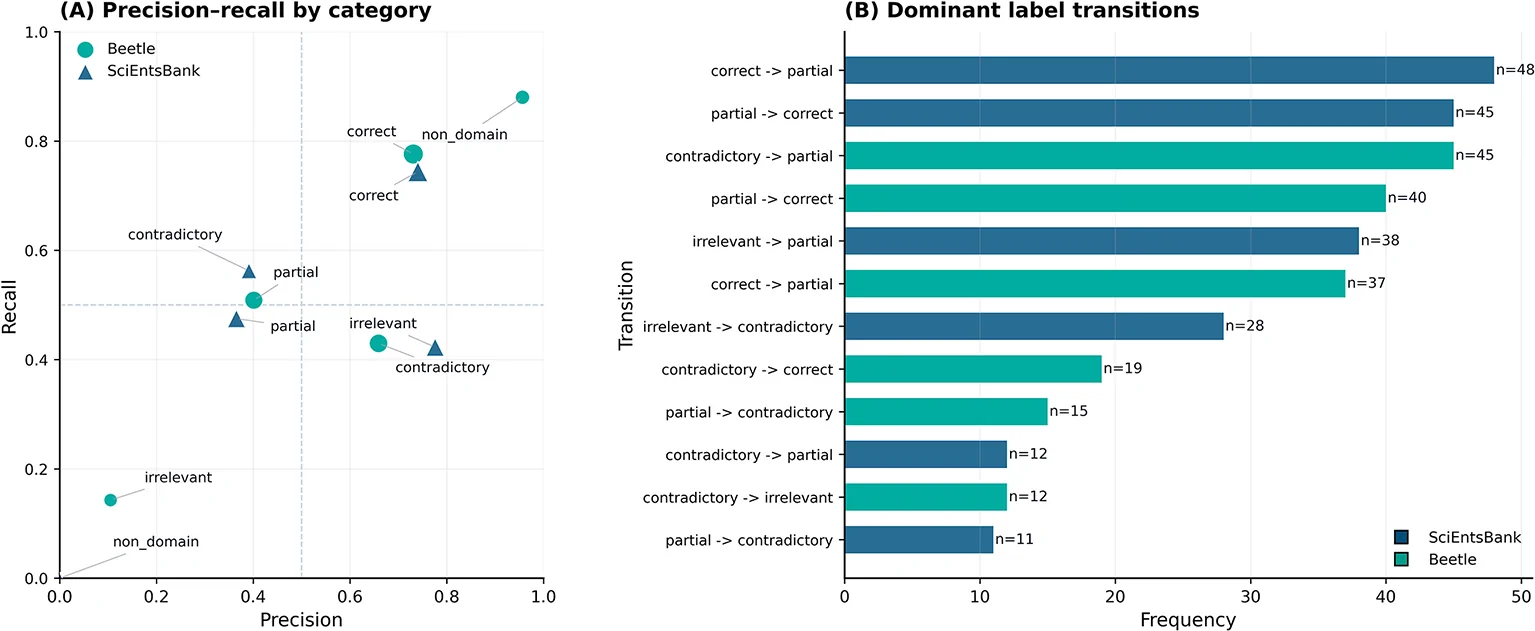
Diagnosis of predictive behavior in short-answer tasks. **(A)** Relationship between precision and recall for each evaluation category in Beetle and SciEntsBank. **(B)** Main transitions between human classification and model prediction.

The distribution of class-level performance can be examined from two complementary perspectives: the balance between Precision and Recall for each evaluation category and the transitions between human annotations and model predictions. As illustrated in Figure 3A, the categories exhibit a clear separation between high- and low-stability regions in the precision-recall space. The *correct* label occupies the most stable region across the short-answer benchmarks, whereas *non–domain* shows high stability only when its empirical support is sufficient. The partially correct/incomplete, contradictory, and irrelevant categories occupy intermediate or lower regions. This distribution confirms that prediction degradation is concentrated in semantically adjacent categories rather than being uniformly distributed across all classes.

Figure 3B further demonstrates that prediction errors are concentrated between semantically adjacent categories rather than between conceptually opposite classes. The transitions *correct*→*partially_correct_incomplete, partially_correct_incomplete*→*correct*, and *contradictory*→*partially_correct_incomplete* account for the largest proportion of misclassifications. This pattern indicates that the language model generally preserves the overall semantic orientation of student responses while lacking the resolution required to distinguish neighboring levels of conceptual understanding. Conversely, the absence of frequent transitions between extreme categories suggests that prediction errors arise from systematic semantic ambiguity rather than from random category assignments.

The recurrence of these transitions reveals stable error mechanisms that extend beyond individual predictions. As summarized in Table 6, the dominant misclassification patterns follow consistent decision rules instead of isolated failures. The five most frequent mechanisms exhibit a common tendency to compress semantic distinctions between adjacent categories, either by shifting correct responses toward partial understanding or by interpreting incomplete evidence as conceptually sufficient. Moreover, the low entropy values associated with all identified mechanisms indicate that these transitions remain highly stable across repeated executions, demonstrating that the observed behavior is not driven by stochastic variability during inference. Collectively, these findings suggest that the principal limitation of the language model is not recognizing domain knowledge itself but maintaining sufficiently precise semantic boundaries to discriminate between closely related levels of conceptual understanding.

**Table 6:** Predominant semantic error mechanisms identified in the short-answer benchmarks.

Mechanism	Benchmark	Dominant transition	Instances	Entropy	Interpretation
Compression of correct answers	SciEntsBank	Correct → partial	48	0.090	Correct responses are downgraded to partial understanding.
Overacceptance of partial answers	SciEntsBank	Partial → correct	45	0.081	Incomplete evidence is interpreted as conceptually sufficient.
Absorption of contradictions	Beetle	Contradictory → partial	45	0.204	Conceptual contradictions are softened into partial understanding.
Technical overacceptance	Beetle	Partial → correct	40	0.054	Technically related responses are accepted as fully correct.
Softening of irrelevance	SciEntsBank	Irrelevant → partial	38	0.115	Off-topic responses acquire unwarranted partial relevance.

#### 4.3.2 Ordinal errors in ASAP2

**Figure 4:**
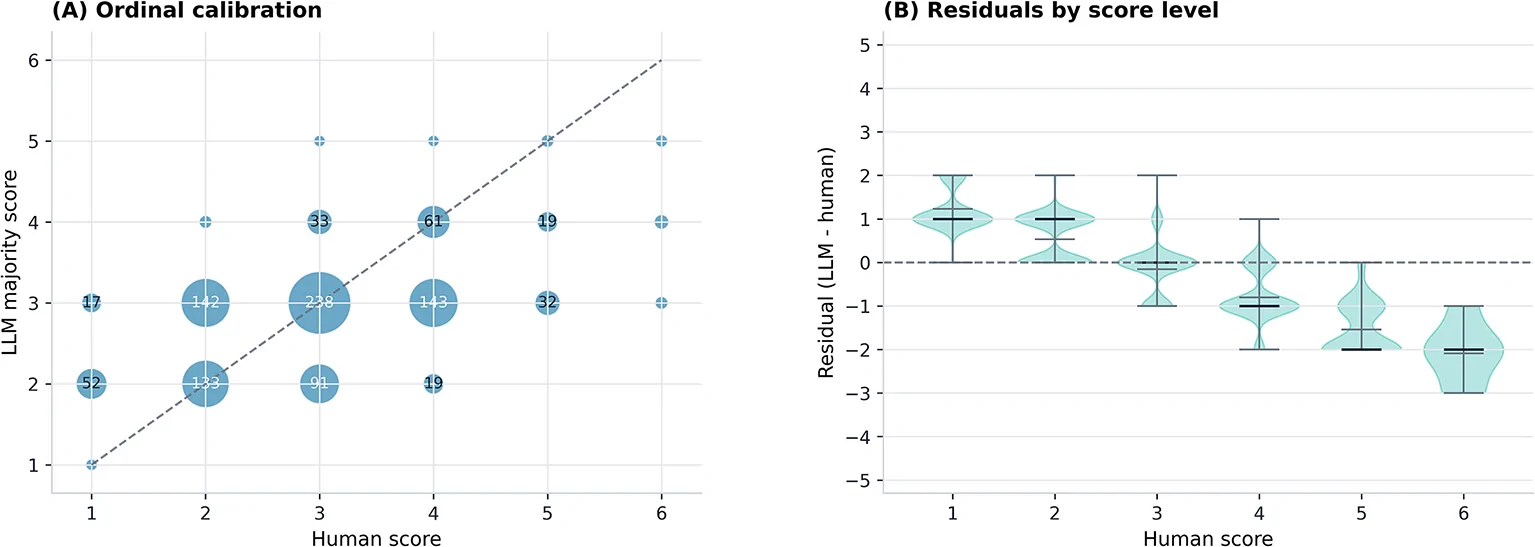
Calibration and residual analysis in ASAP2. **(A)** Relationship between human score and majority model score. **(B)** Distribution of residuals by ordinal scale level.

Automated essay scoring requires preserving not only the proximity between predicted and human ratings but also the ordinal structure of the evaluation scale. Consequently, satisfactory predictive performance cannot be interpreted exclusively through average error measures, since systematic shifts toward particular score levels may reduce absolute errors while simultaneously diminishing the model's ability to discriminate between essays of substantially different quality. Figure 4 characterizes this behavior by jointly examining prediction calibration and the distribution of residual errors.

As illustrated in Figure 4A, the highest concentration of predictions occurs around the intermediate levels of the rating scale, largely independent of the original human annotation. Essays assigned lower human scores tend to receive comparatively higher predictions, whereas essays with higher ratings exhibit the opposite tendency. Consequently, model predictions progressively converge toward the center of the ordinal scale, compressing the effective separation between adjacent performance levels. This calibration pattern indicates that the language model preserves the overall ranking of essay quality while reducing its sensitivity to distinguish between the lowest- and highest-quality responses.

This calibration pattern explains the coexistence of a relatively low MAE and a moderate QWK reported in the previous subsection. Most prediction discrepancies correspond to shifts of a single score level, thereby limiting the average absolute error. Nevertheless, these systematic shifts progressively distort the ordinal structure of the rating scale and reduce overall agreement with human assessment. Consequently, the principal limitation of the language model is not the production of extreme scoring errors but the reduced ability to discriminate consistently between adjacent levels of writing quality.

Figure 4B demonstrates that this behavior arises from a systematic calibration bias rather than from stochastic variability during inference. Lower score levels are characterized predominantly by positive residuals, indicating a tendency to overestimate lower-quality essays, whereas higher score levels exhibit negative residuals associated with underestimation. Rather than being symmetrically distributed around zero, the residuals converge progressively toward the center of the scale, providing clear evidence of an ordinal compression process.

The regularity of this residual distribution indicates that the observed behavior affects the entire rating scale rather than isolated score levels. No abrupt discontinuities or localized regions of instability are observed. Instead, prediction errors follow a gradual convergence toward intermediate categories, progressively reducing the effective separation between the lowest-, middle-, and highest-quality essays. This finding suggests that the reduction in predictive performance is driven by a systematic ordinal prediction mechanism instead of benchmark-specific artifacts or isolated inference failures.

The recurrence of these ordinal transitions can be summarized through a small set of interpretable prediction mechanisms. As reported in Table 7, the five dominant transition patterns exhibit a highly structured distribution. The two most frequent mechanisms (*score 4*→*score 3* and *score 2*→*score 3*) together account for almost half of all dominant transitions, indicating that the central level of the rating scale acts as an attractor for model predictions. Consequently, ordinal prediction errors are not uniformly distributed across score levels but instead reflect a systematic compression of the effective scoring range.

**Table 7:** Predominant ordinal prediction mechanisms identified in ASAP2.

Mechanism	Dominant transition	Instances	Interpretation
Downward compression	Score 4 → Score 3	143	High-quality essays shift toward intermediate score levels.
Upward compression	Score 2 → Score 3	142	Lower-quality essays are systematically elevated toward the center of the scale.
Compression of intermediate levels	Score 3 → Score 2	91	The separation between neighboring intermediate categories remains unstable.
Upward elevation of lower levels	Score 1 → Score 2	52	The weakest essays rarely remain at the lowest score level.
Attenuation of upper levels	Score 5 → Score 3	32	Higher-quality essays lose differentiation from intermediate essays.

The remaining mechanisms reinforce this interpretation. Both initial overestimation and upper underestimation show that the extremes of the scale are less stable than the central categories. Therefore, low-quality essays tend to receive more favorable scores, while higher-quality essays lose some of the differentiation achieved by human evaluators. The evidence indicates that the model adequately preserves the overall order among responses but reduces the resolution required to accurately reproduce the ordinal separation between performance levels.

### 4.4 Structural quality of LLM-generated feedback

The structural quality of the feedback generated by the language model varied substantially across educational assessment tasks. Whereas essay scoring produced highly structured feedback explicitly oriented toward student improvement, short-answer assessment generated considerably shorter explanations with more limited pedagogical content. As summarized in Table 8, ASAP2 achieved the highest overall feedback quality index (0.973), clearly exceeding the values observed for Beetle (0.681) and SciEntsBank (0.693). Similarly, ASAP2 produced the highest levels of specificity (0.896) and constructiveness (0.917), indicating that richer textual contexts facilitate the generation of more detailed explanations, stronger pedagogical recommendations, and more explicit justification of assessment decisions.

**Table 8:** Structural quality indicators of the feedback generated across the educational benchmarks.

Benchmark	Feedback Index	Specificity	Constructiveness	Median words	Interpretation
ASAP2	0.973	0.896	0.917	97	Highly structured feedback with detailed pedagogical guidance.
Beetle	0.681	0.635	0.060	22	Correct assessments but limited explanatory and constructive content.
SciEntsBank	0.693	0.657	0.144	22	Relevant feedback with modest pedagogical development.

Conversely, the three benchmarks demonstrate that the amount of contextual evidence available to the language model is one of the principal factors influencing feedback quality. In ASAP2, the median feedback length reached 97 words, providing sufficient space to integrate explanations, supporting arguments, and pedagogical recommendations within a single response. In contrast, Beetle and SciEntsBank produced a median of only 22 words, substantially limiting the opportunity to elaborate detailed educational guidance. This difference extends beyond the length of the generated text itself and reflects the amount of contextual information available to support meaningful pedagogical explanations.

The relationship between specificity and constructiveness provides further evidence of this limitation. Although specificity remains relatively stable across the short-answer benchmarks (0.635 for Beetle and 0.657 for SciEntsBank), constructiveness decreases substantially (0.060 and 0.144, respectively). These results indicate that the language model generally identifies the central concept addressed by the student response but encounters greater difficulty translating that assessment into actionable recommendations for learning improvement. Consequently, the principal limitation of feedback in short-answer assessment lies not in recognizing relevant content but in generating pedagogically useful explanations from inherently limited contextual evidence.

**Figure 5:**
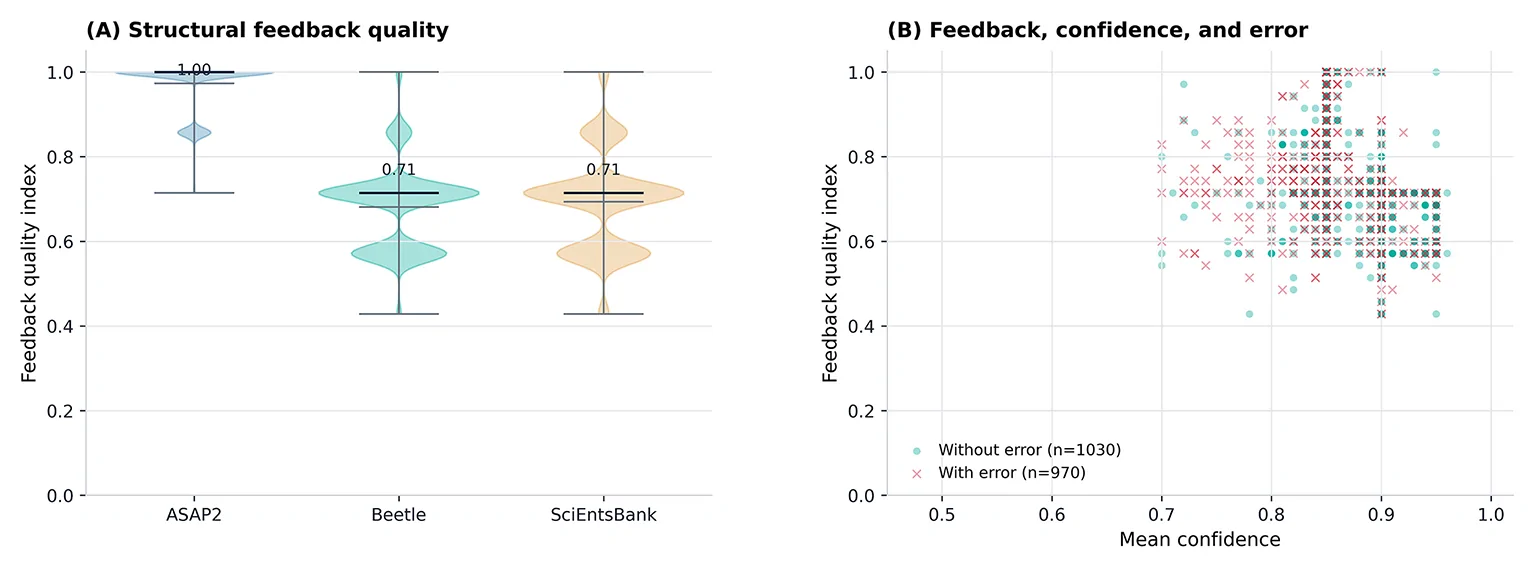
Structural quality of the generated feedback. **(A)** Distribution of the feedback quality index by benchmark. **(B)** Relationship between confidence in the prediction, the feedback quality index, and the presence of classification errors.

The stability of these structural indicators can be further examined through the distribution of the feedback quality index and its relationship with prediction confidence. As illustrated in Figure 5A, ASAP2 exhibits the most homogeneous distribution, with feedback quality values consistently concentrated near the upper end of the scale. In contrast, Beetle and SciEntsBank present substantially broader distributions shifted toward lower values, indicating greater variability in the quality of the generated explanations. These findings suggest that language models maintain more consistent explanatory behavior when evaluating extended argumentative texts, whereas feedback quality becomes increasingly heterogeneous as the amount of contextual information available for assessment decreases.

Figure 5B further demonstrates that prediction confidence and the structural quality of LLM-generated feedback exhibit only a partial correspondence. Numerous observations combine high confidence with both correct and incorrect classifications while producing feedback of comparable structural quality. This distribution indicates that the linguistic quality of generated explanations represents a property that is only partially associated with predictive accuracy. Consequently, well-structured feedback does not necessarily imply a correct assessment, just as an incorrect prediction may still be accompanied by coherent, pedagogically meaningful, and potentially useful explanations for the student.

The practical implications of this relationship become evident through the representative cases presented in Table 9. These examples confirm that the pedagogical value of generated feedback cannot be inferred exclusively from classification accuracy. Cases SA-1 and ES-1 illustrate situations in which both the prediction and the generated feedback remain fully consistent with the corresponding human assessment, producing recommendations that appropriately support student learning. Conversely, SA-2 represents one of the most critical situations identified during the evaluation, where an incorrect prediction made with high confidence is accompanied by a coherent explanation that reinforces a conceptually incorrect interpretation. In contrast, ES-2 and ES-3 demonstrate that small ordinal deviations do not necessarily compromise the educational usefulness of the generated feedback. Even when the assigned score differs from the human reference, the language model continues to identify relevant strengths, weaknesses, and recommendations that may still contribute to student improvement.

**Table 9:** Representative cases illustrating the relationship between predictive performance and feedback quality.

Case	Scenario	Representative excerpt	Interpretation
SA-1	Correct classification	“...the terminals are not connected...”	Prediction and feedback remain fully consistent with the human reference.
SA-2	High-confidence error	“...closed circuit...”	A confident but incorrect prediction is accompanied by a coherent explanation supporting an erroneous interpretation.
SA-3	Ambiguous response	“...bulb C is out...”	The feedback identifies relevant concepts but loses the specific context required for the assessment.
ES-1	Correct essay assessment	Essay on Venus	Complete agreement between the assigned score and the generated feedback.
ES-2	Minor ordinal deviation	Essay on Seagoing Cowboys	The feedback remains pedagogically useful despite a slight score overestimation.
ES-3	Ordinal overestimation	Essay on Venus	Relevant strengths and weaknesses are identified even though the assigned score differs from the human reference.

## 5 Discussion

The present findings are consistent with recent studies arguing that the educational adoption of LLMs should be evaluated beyond their ability to generate plausible responses. Recent work emphasizes that the principal challenge is no longer integrating LLMs into educational workflows but establishing reliable, transparent, and reproducible evaluation procedures capable of supporting assessment decisions (Yan et al., 2024; Emirtekin, 2025; Saez et al., 2026). The results obtained with EduFairBench support this perspective by demonstrating that predictive performance, robustness, uncertainty, and feedback quality provide complementary evidence that cannot be adequately represented by any single evaluation metric.

The principal novelty of EduFairBench is methodological integration. Majority voting, bootstrap confidence intervals, repeated inference, uncertainty estimation, and error analysis are established techniques, but they are frequently reported as isolated analyses or omitted from LLM-based educational assessment studies. EduFairBench combines these components into a traceable protocol in which each dataset instance, prompt version, model execution, parsed output, consolidated prediction, metric, table, and figure can be linked within the same reproducible workflow. This design directly addresses concerns identified in the recent literature, including incomplete prompt reporting, insufficient dataset documentation, weak reproducibility, and limited uncertainty-aware evaluation.

The experimental results further suggest that the behavior of language models is determined primarily by the characteristics of the assessment task rather than by the educational domain alone. Although the two short-answer benchmarks achieved comparable levels of overall Accuracy, their class-level analyses revealed substantially different discrimination capabilities across semantic categories. In contrast, the essay benchmark preserved moderate ordinal agreement while maintaining relatively small deviations between predicted and human scores. These observations indicate that identical aggregate performance values may conceal fundamentally different prediction behaviors depending on whether the evaluation task requires nominal categorization or ordinal judgment.

A second contribution of this study concerns the interpretation of robustness. The repeated inference protocol demonstrated that highly reproducible predictions may coexist with systematic prediction errors, high confidence, and low entropy. This distinction is consistent with recent evidence showing that reliability and accuracy do not necessarily co-vary in LLM-based educational judgment (Shin, 2026). In EduFairBench, the majority-vote analysis further showed that consolidation did not substantially increase performance relative to individual executions. Its value was therefore methodological: it produced a stable, auditable prediction while preserving indicators of disagreement, entropy, and confidence for interpretation.

The evaluation of LLM-generated feedback provides an additional perspective that remains comparatively underexplored in current educational assessment research. The results demonstrate that the structural quality of feedback depends strongly on the amount of contextual evidence available to the model. More importantly, representative cases show that coherent and pedagogically plausible explanations may accompany incorrect predictions, whereas small scoring discrepancies do not necessarily reduce the potential usefulness of formative comments. This finding supports recent calls to evaluate feedback quality separately from scoring agreement (Saez et al., 2026; Kim, 2025). In the present study, however, feedback quality was assessed through objective structural indicators rather than through teacher or student ratings. Therefore, the results should be interpreted as evidence of structural completeness and pedagogical orientation, not as evidence of classroom effectiveness. This distinction limits the interpretation of the feedback results to reproducible output characteristics and avoids making claims about learner uptake, teacher acceptance, or formative impact in authentic classrooms.

Several limitations should be considered. First, the empirical instantiation used a single model, GPT-4.1-mini, with temperature 0.2 and a maximum output length of 900 tokens. EduFairBench is model-independent at the protocol level, but the reported results should not be interpreted as a comparison among LLMs or as evidence of superiority over Claude, Gemini, Llama, DeepSeek, or other systems. Second, the benchmarks provide standardized and reproducible assessment conditions, but they do not fully represent authentic classroom assessment, university term papers, teacher-mediated feedback, or contemporary learning environments in which students may interact with generative AI during the production of their responses. Generalization to these settings requires additional validation with real instructional tasks, institutional rubrics, teacher judgments, student responses to feedback, and classroom implementation data. Third, because the study did not include human evaluation of generated feedback, interrater agreement for feedback quality was not applicable.

Overall, the present results demonstrate that the evaluation of educational LLMs benefits from a multidimensional methodological perspective extending beyond conventional performance metrics. Rather than proposing new predictive metrics or replacing existing evaluation practices, EduFairBench complements current methodologies by integrating repeated inference, prediction consolidation, uncertainty estimation, semantic error analysis, and structural feedback evaluation within a single reproducible experimental workflow. This integrated perspective facilitates more meaningful comparisons across educational benchmarks and provides a reusable methodological framework for rigorous evaluation of current and future language models in open-ended educational assessment.

## 6 Conclusions

This study presented EduFairBench, a methodological framework for the reproducible evaluation of large language models in open-ended educational assessment. Rather than proposing a new predictive model or introducing alternative performance metrics, EduFairBench provides a unified experimental protocol that integrates repeated inference, prediction consolidation, uncertainty estimation, semantic error analysis, and structural evaluation of LLM-generated feedback within a fully traceable workflow. This multidimensional design enables a more comprehensive characterization of language model behavior than conventional evaluation approaches based exclusively on aggregate performance measures.

The experimental results demonstrate that educational LLMs cannot be adequately characterized using predictive accuracy alone. Predictive agreement, inference robustness, uncertainty, semantic error patterns, and feedback quality capture complementary properties of model behavior that evolve differently across assessment tasks. In particular, the study shows that highly reproducible predictions may coexist with systematic classification errors, while pedagogically coherent feedback can accompany imperfect predictions. These findings highlight the importance of evaluating multiple dimensions simultaneously in order to obtain a realistic understanding of language model performance in educational assessment.

Beyond the experimental validation performed on three publicly available educational benchmarks, the principal contribution of this work lies in establishing a reusable evaluation methodology that is independent of any specific benchmark or language model. Because the protocol separates the experimental workflow from the predictive system being evaluated, it can be directly applied to alternative datasets, educational domains, prompting strategies, and future generations of language models. This generality facilitates more consistent comparisons across studies while supporting transparent, traceable, and scientifically rigorous evaluation practices within educational artificial intelligence.

Future work should extend EduFairBench toward comparative evaluations involving multiple language models, prompting strategies, reasoning configurations, and authentic classroom assessment contexts while incorporating human-centered validation of generated feedback through teachers and students. These extensions would strengthen empirical evidence regarding AI-assisted educational assessment and contribute to the continued development of standardized, reproducible evaluation methodologies for the responsible integration of language models into educational practice.

## Data Availability

Publicly available datasets were analyzed in this study. SciEntsBank and Beetle are publicly available as part of the SemEval-2013 Task 7 Student Response Analysis benchmark, as documented in the Association for Computational Linguistics (ACL) Anthology: https://aclanthology.org/S13-2045/. The datasets are currently accessible through the following public repositories: SciEntsBank: https://huggingface.co/datasets/nkazi/SciEntsBank; Beetle: https://huggingface.co/datasets/nkazi/BeetleASAP2. ASAP 2.0 (Automated Student Assessment Prize 2) is publicly available through Kaggle: https://www.kaggle.com/datasets/lburleigh/asap-2-0.
